# Preliminary Validation of a Food Frequency Questionnaire to Assess Long-Chain Omega-3 Fatty Acid Intake in Eye Care Practice

**DOI:** 10.3390/nu11040817

**Published:** 2019-04-11

**Authors:** Alexis Ceecee Zhang, Laura E. Downie

**Affiliations:** Department of Optometry and Vision Sciences, The University of Melbourne, Parkville, 3010 Victoria, Australia; alexisz@student.unimelb.edu.au

**Keywords:** omega-3, fatty acid, diet, dietary assessment, clinical survey, eye disease, dry eye, age-related macular degeneration, food frequency questionnaire, CODS

## Abstract

Clinical recommendations relating to dietary omega-3 essential fatty acids (EFAs) should consider an individual’s baseline intake. The time, cost, and practicality constraints of current techniques for quantifying omega-3 levels limit the feasibility of applying these methods in some settings, such as eye care practice. This preliminary validation study, involving 40 adults, sought to assess the validity of a novel questionnaire, the Clinical Omega-3 Dietary Survey (CODS), for rapidly assessing long-chain omega-3 intake. Estimated dietary intakes of long-chain omega-3s from CODS correlated with the validated Dietary Questionnaire for Epidemiology Studies (DQES), Version 3.2, (Cancer Council Victoria, Melbourne, Australia) and quantitative assays from dried blood spot (DBS) testing. The ‘method of triads’ model was used to estimate a validity coefficient (ρ) for the relationship between the CODS and an estimated “true” intake of long-chain omega-3 EFAs. The CODS had high validity for estimating the ρ (95% Confidence Interval [CI]) for total long-chain omega-3 EFAs 0.77 (0.31–0.98), docosahexaenoic acid 0.86 (0.54–0.99) and docosapentaenoic acid 0.72 (0.14–0.97), and it had moderate validity for estimating eicosapentaenoic acid 0.57 (0.21–0.93). The total long-chain omega-3 EFAs estimated using the CODS correlated with the Omega-3 index (*r* = 0.37, *p* = 0.018) quantified using the DBS biomarker. The CODS is a novel tool that can be administered rapidly and easily, to estimate long-chain omega-3 sufficiency in clinical settings.

## 1. Introduction

Omega-3 polyunsaturated fatty acids (PUFAs) are essential fatty acids (EFAs) that cannot be synthesized de novo, and thus must be derived from food sources or dietary supplementation. The potential benefit of diets rich in omega-3 fatty acids has been shown in a variety of health conditions, such as hypercholesterolaemia and rheumatoid arthritis [1,2]. Omega-3 PUFAs exist in both short- and long-chain forms. The short-chain omega-3 fatty acids and alpha-linoleic acid (ALA) are derived from plant-based sources (e.g., flaxseed and walnuts) and they are a precursor to the more biologically potent long-chain omega-3 EFAs, docosahexaenoic acid (DHA) and eicosapentaenoic acid (EPA). Dietary long-chain omega-3 EFAs are found mostly in marine sources (e.g., oily fish and seafood). Once ingested, long-chain omega-3 PUFAs are incorporated into cellular membranes and play a role in cellular signaling, modulating systemic inflammation, and influencing immune function [3,4]. 

There is mounting evidence that diets rich in omega-3 EFAs may be beneficial for reducing the risk of development and/or progression of several ocular conditions, such as dry eye disease and age-related macular degeneration (AMD) [5,6,7]. The United States (US) Women’s Health Study showed that a low dietary intake of omega-3 EFAs is associated with a higher incidence of dry eye disease in women [7]. The Blue Mountains Eye Study found that eating oily fish once per week, as compared with fewer than once per week, was associated with a lower risk of developing early-stage AMD [8]. A meta-analysis of prospective cohort, case-control, and cross-sectional studies suggested that consumption of two or more servings of oily fish per week was beneficial in the primary prevention of AMD [9]. An association has been found between higher dietary intake of omega-3 EFAs and significant risk reduction for developing more advanced, sight-threatening forms of AMD [10,11,12]. Omega-3 EFA supplementation may also lower intraocular pressure in adults [13].

Currently, the suggested dietary targets (SDT) for long-chain omega-3 EFA consumption, as recommended by the Australian National Health and Medical Research Council (NHMRC), in diets optimized to lower chronic disease risk, is 430 mg/day for women and 610 mg/day for men [14,15]. These recommendations are consistent with the National Heart Foundation position statement (2015) that recommends two to three servings of fish (serving size: 150–200 g), preferably oily fish, per week to achieve ~250–500 mg/day of combined EPA and DHA consumption [16]. The US Department of Health and Human Services Dietary Guidelines (2015–2020) recommend eight ounces of fish/seafood per week, which is approximately equivalent to 250 mg of EPA + DHA per day, and eight to 12 ounces in pregnancy [17]. These recommended values are similar to that of the European Food Safety Authority [18], while the French Agency for Food, Environmental and Occupational Health and Safety (ANSES) recommends 250 mg/day of each EPA and DHA. However, research suggests that approximately 80% of Australian adults do not meet this recommendation for daily intake [19]. Only ~15% of the French population meet the recommendation for daily intake of EPA, and 8% are estimated to meet the daily DHA recommendation. A study undertaken in the US estimated that low dietary intake of omega-3 fatty acids was a modifiable risk factor contributing to ~84,000 deaths in 2005 [20]. Therefore, there is a need for healthcare clinicians to actively enquire about their patients’ diet, and preferably quantify their dietary intake of omega-3 EFAs, in order to identify individuals who are likely to benefit from dietary changes and/or supplementation to improve their health.

Various techniques can be used to quantify systemic omega-3 EFA levels, however, most of these methods are not ideally suited for direct application in all clinical settings [21]. Fatty acid levels present in subcutaneous adipose tissue are considered to be the most robust long-term marker of fatty acid intake [22]. Systemic EFA concentrations can be accurately estimated by assaying fatty acid levels in erythrocytes and plasma phospholipids [23]. Although both of these methods provide quantitative data relating to systemic fatty acid levels, the sample collection procedures are invasive, relatively costly, and time consuming (as they require off-site laboratory analyses), and therefore are not currently routinely performed in a clinical setting [24]. In addition, these tests are not readily accessible to all clinicians who may be in a position to provide relevant dietary advice regarding the potential benefits of omega-3 EFA intake to their patients (e.g., eye care clinicians, in relation to ocular health). Short-term dietary assessment, such as dietary records requiring self-monitored and detailed recordings over multiple days, and short-term recalls do not account for the day-to-day variation of a habitual diet [25]. Furthermore, both methods have been shown to underestimate average energy intake, particularly in populations with lower socioeconomic status, education, and literacy levels [26]. 

As an alternative, diet questionnaires provide a rapid, non-invasive method for estimating dietary fatty acid intake. Although nutrient intakes derived from several food frequency questionnaires (FFQs) have been shown to correlate with quantitative biological markers of omega-3 EFA intake [21], questionnaires designed for use in epidemiology research studies are often exhaustive and relatively time consuming, and not intended for use in routine clinical settings [27,28]. Several brief omega-3 dietary questionnaires have been developed for use in clinical settings [29], including for adults with psychological disorders [30,31], and to use for identifying older adults with inadequate omega-3 EFA intake in the context of cardiovascular disease risk [32]. The reliability estimates from different dietary surveys are dependent on the nutritional composition of food sources, which can vary across different geographical locations [33].

The aim of this study was to undertake preliminary validation of a novel, clinically applicable questionnaire, the Clinical Omega-3 Dietary Survey (CODS), for assessing dietary long-chain omega-3 EFA intake, as compared with an objective fatty acid blood biomarker and a previously validated survey designed to capture a full dietary profile. Information derived from this tool could then be used by clinicians to estimate whether an individual meets the recommended daily intake of long-chain omega-3 EFAs, or whether they might benefit from enhancing their dietary intake.

## 2. Materials and Methods 

This cross-sectional research study was conducted in accordance with the tenets of the Declaration of Helsinki, and it was approved by the University of Melbourne Human Research Ethics Committee (HREC #1749830.1) and the St Vincent’s Hospital Research Ethics Committee (HREC #HREC/17/SVHM/236). 

### 2.1. Participants

Adult participants were recruited from the University of Melbourne and St Vincent’s Hospital (Melbourne, Victoria, Australia) via advertisements. All participants provided written, informed consent to participate. Eligible participants were aged 18 years or older, and were not pregnant or breastfeeding. There were no eligibility restrictions with respect to general health conditions. Long-chain omega-3 EFA status was quantified using three methods, as detailed below: (a) the CODS; (b) the Dietary Questionnaire for Epidemiological Studies, Version 3.2 (DQES v3.2, Cancer Council Victoria, Australia); and (c) dried blood spot (DBS) testing (Xerion Pty Ltd, Victoria, Australia). For methods (a) and (b), daily long-chain omega EFA intake (mg/day) was calculated as the sum of EPA, DHA, and docosapentaenoic acid (DPA) intake. For method (c), the long-chain omega-3 EFAs present in erythrocytes (%) was calculated as the sum of EPA, DHA, and DPA concentrations (%) present in erythrocytes.

### 2.2. Clinical Omega-3 Dietary Survey

A clinically relevant FFQ, the CODS, was developed by the authors of this study to yield an estimate of long-chain omega-3 EFA intake, using the nutrient composition data available in the AUSNUT (2011–2013) food nutrient database (available online at: http://www.foodstandards.gov.au/science/monitoringnutrients/ausnut/foodnutrient/Pages/default.aspx). For natural foods containing the highest concentrations of total long-chain omega-3 fatty acids (ranging from 769.4 mg/100 g to 62 mg/100 g), the fatty acid composition was extracted from the nutritional database, and the food was considered for inclusion in the CODS. 

The CODS (Figure S1) comprises three main sections, categorized according to the primary type of food as: (i) seafood, (ii) fish, and (iii) meat (including eggs). Seven species of seafood, 17 types of fish (including a generalized description for white-fleshed fish), and seven different types of meat/eggs are incorporated, and are used as a basis for estimating dietary long-chain omega-3 EFA intake. A final section, (iv), considers the consumption of long-chain omega-3 EFAs from dietary supplements. The study participants were required to provide details relating to the supplement formulation (brand, dose, form of omega-3 EFAs, and frequency of intake) and these data were incorporated into the analysis. 

The CODS was administered by a single examiner (A.C.Z.), and the participants’ responses were recorded on a paper-based survey. The first part of the survey asked participants to recall the average frequency (i.e., number of times per week or per month, as appropriate for the nominated frequency) of fish or seafood consumption (in any form, including fresh and canned) over the past three months. The method provided to the participants for estimating the approximate portion size (in amounts of 50 g, 100 g, 150 g and 200 g) required using the size of the examiner’s palm as a reference for a 100 g portion size. Then, the participants were required to estimate the average portion size (in grams) of fish or seafood they typically consumed. 

For each food source listed in sections (i), (ii), and (iii) of the CODS, participants were asked to estimate the average portion size per serving (g), and to note how often they consumed that type of food (i.e., average number of times per week or per month). In order to quantify egg intake, the average number of eggs per week or per month was reported. The participants were instructed to only include food sources that they consumed “regularly”, defined as at least once per month over the past three months. 

Quantification of the total long-chain omega-3 EFA intake was derived from the composition data available in the AUSNUT (2011–2013) food nutrient database [34], using a spreadsheet in Microsoft Excel 2017 (Microsoft Corp., Redmond, WA, USA).

### 2.3. Dietary Questionnaire for Epidemiology Studies

An online diet questionnaire, DQES v3.2, was purchased from an independent provider (Cancer Council Victoria, Australia) [35]. The DQES v3.2 was developed and validated for assessing intake of food and nutrients among Australian adults [36,37], and it had been previously utilized by the Melbourne Collaborative Cohort epidemiology study, involving a cohort of 810 participants. 

The DQES v3.2 consisted of 142 food items, and it used extensive food compositional data derived from two Australian databases, AUSNUT 2007 and NUTTAB 2010 [38,39], to estimate dietary intake for a spectrum of 98 nutrients, including macronutrients (e.g., carbohydrates, proteins, fats) and micronutrients (e.g., vitamins, minerals). In addition, nutrient intakes from alcoholic beverages are surveyed, although these values were not incorporated into the analysis.

Nutritional information relating to EFAs was estimated from the questionnaire, and included a breakdown of individual fatty acids, derived from the NUTTAB 2010 food database [39]. The total long-chain omega-3 EFA intake (mg/day) was calculated as the sum of P205W3FD (EPA), P225W3FD (DPA), and P226W3FD (DHA). As the DQES is proprietary, we were unable to ascertain which specific food items were used to derive the estimate of omega-3 fatty acid intake for this survey.

Participants undertook the DQES online, using a self-administrated delivery method. Once completed, the nutritional analysis for each participant was undertaken by the survey supplier, with the results e-mailed to the study authors. 

### 2.4. Dried Blood Spot Testing

The participants’ fatty acid profiles were analyzed using dried DBS testing (PUFAcoat™ technology, Xerion Pty Ltd, Victoria, Australia). A single drop of capillary blood (~3 mL) was collected from each participant, using a sterile, single-use lancing device and spotted onto the proprietary PUFAcoat™ test cards, which are designed for long-term stabilization of long-chain PUFAs in dried blood samples. Prior to analysis, the DBS cards were air dried, placed in sealed cellophane bags, and stored in desiccants in a dark, temperature-controlled chamber. As per the manufacturer’s instructions, all blood samples were sent for laboratory analyses within four weeks of collection. 

The fatty acid analyses were performed in an independent laboratory (Waite Lipid Analysis Service (WLAS), University of Adelaide, South Australia, Australia), using established methods [40]. In brief, the fatty acid blood spots were transmethylated by mixing with H_2_SO_4_ (18M AR grade, BDH, Sussex, UK) in anhydrous methanol in a 5 mL sealed vial (Wheaton, Millville, USA), and then were heated for three hours at 70 °C. The fatty acid methyl esters were separated and quantified using gas chromatography (Hewlett-Packard 6890 gas chromatograph, equipped with a 50 m capillary column (0.32 mm internal diameter SGE, Victoria), coated with 70% cyanopropyl polysilphenylene-siloxane (BPX70) (0.25 μm film thickness) and fitted with a flame ionization detector. A helium carrier gas was used and the inlet split ratio was set to 20:1, with the injector temperature at 250 °C and detector temperature at 300 °C. Fatty acid methyl esters were identified by comparing the retention times and the peak area values of unknown samples to the standards using the ChemStation software (Hewlett Packard, CA, USA), and a normalized percentage was calculated based on the response factors. 

The extracted long-chain omega-3 fatty acid output parameters were: 22:5n-3 (EPA), 22:5n-3 (DPA) and 22:6n-3 (DHA). The total long-chain omega-3 EFA erythrocyte concentration (%) was calculated as the sum of: C20:5n-3 (EPA) + C22:5n-3 (DPA) + C22:6n-3 (DHA). The Omega-3 index (%) was defined as the total percentage of EPA and DHA present in erythrocyte phospholipid membranes [40]. 

### 2.5. Statistical Analysis

On the basis of an estimated correlation coefficient of 0.45 between quantification methods, as previously reported in a similar study [41], a sample size of 36 participants was calculated to be required for 80% power at a 5% significance level. 

Data normality testing was performed using the D’Agostino–Pearson omnibus normality test. The inter-group comparisons were analyzed using a Student’s t-test or Mann–Whitney U test, as appropriate. The Spearman’s correlation coefficient (r_s_) was used to assess the relationship between outputs from the CODS, DQES, and DBS analyses, with respect to long-chain omega-3 EFA intake levels. The Spearman’s correlation coefficients were interpreted with reference to similar studies, where r_s_ ≤ 0.35 indicates a weak correlation, r_s_ = 0.36–0.67 indicates a moderate correlation, r_s_ = 0.68–1 indicates a good correlation and r_s_ ≥ 0.9 indicating a very good correlation [28].

The ‘method of triads’ statistical model was used to derive a validity coefficient to estimate the relationship between a dietary measurement (e.g., the CODS) and an estimated “true” intake (Figure S2) [42,43]. Validity coefficients closer to 1 indicated a closer relationship between the estimated dietary score and the estimated “true” intake [27]. For this analysis, a validity coefficient <0.2 was considered to represent low validity, values between 0.2 and 0.6 were considered to represent moderate validity, and values >0.6 were regarded as indicative of high validity [43]. For a Heywood case, where a validity coefficient ≥1 was estimated, the validity coefficient was set to 1.

A bootstrap procedure was used to estimate the confidence interval (CI) for each validity coefficient [43,44]. For this method, each bootstrap drew 40 samples with replacement from the original sample, and the validity coefficient for each quantification method was calculated. A total of 1000 bootstrap samples were obtained to build a bootstrap distribution of validity coefficients using Microsoft Excel 2017 (Microsoft Corp., Redmond, WA, USA). The non-Heywood cases were used to calculate the 95 percentile CI for each validity coefficient.

The inter-method agreement, for the estimated daily total long-chain omega-3 EFA intake (mg/day) between the CODS and DQES, was examined using Bland–Altman analysis [45]. The mean difference (bias) and limits of agreement (LoA, defined as the bias ±1.96 standard deviations of the mean difference) were calculated. A regression analysis was used to analyze the potential relationship between the differences between the methods and the average of the two methods.

Group data are reported as the mean ± standard deviation (SD), unless otherwise specified.

## 3. Results

Forty participants were enrolled in the study, and they completed three independent methods for assessing long-chain omega-3 EFA intake. The mean (SD) age of participants was 45.7 (18.11) years. Among the 40 participants, 26 were female and 14 were male. The study cohort included individuals with diabetes mellitus (*n* = 19), however, there were no statistically significant differences between this subgroup of participants and the remaining subgroup, with respect to age, gender, or Omega-3 index measured using the DBS biomarker (*p* > 0.05 for each comparison). 

### 3.1. Dietary Assessment of Long-Chain Omega-3 EFA Intake

The average time taken to conduct the CODS was three minutes and the average time taken to complete the DQES (v3.2) was 15 minutes.

Table 1 summarizes the estimated daily long-chain omega-3 EFA intake (mg/day), for each of DHA, DPA, and EPA, as assessed using the CODS and DQES (v3.2), for the study cohort (*n* = 40 participants). There was no significant inter-method difference for the estimated intake of omega-3 fatty acids (Table 1, *p* > 0.05 for all comparisons).

### 3.2. Dried Blood Spot Analysis of Long-Chain Omega-3 EFAs Levels

Table 2 summarizes the estimated long-chain omega-3 EFAs present in erythrocytes, and the Omega-3 index as estimated using the dried blood spot test, for the study cohort. 

### 3.3. Correlation between Quantification Methods

Table 3 summarizes the Spearman’s correlation coefficients (r_s_) for the association between the dietary survey methods (CODS and DQES v3.2, mg/day, Table 1) and DBS assays (%, Table 2) for quantifying DHA, DPA, EPA, and total long-chain omega-3 EFAs (DHA + DPA + EPA).

**Figure 1:**
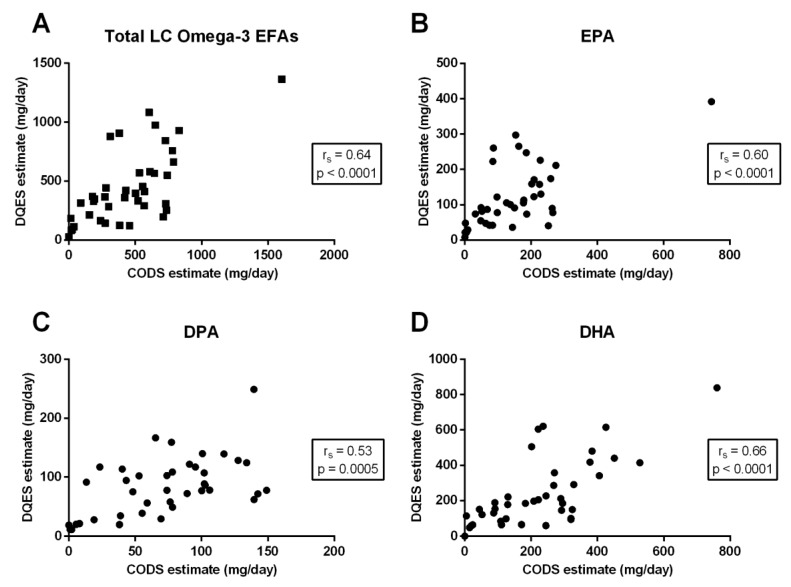
The relationship between calculated dietary long-chain (LC) omega-3 essential fatty acid (EFA) intake, quantified using the Spearman’s correlation coefficients (r_s_), and the Clinical Omega-3 Dietary Survey (CODS) versus the Dietary Questionnaire for Epidemiology Studies (DQES) v3.2, for (**A**) total LC omega-3 EFAs; (**B**) eicosapentaenoic acid (EPA); (**C**) docosapentaenoic acid (DPA); and (**D**) docosahexaenoic acid (DHA).

Comparing the two dietary methods (CODS and DQES v3.2) there was a moderate-to-strong, positive correlation for each of total long-chain omega-3 EFAs (r_s_ = 0.64; *p* < 0.0001) (Figure 1A), EPA (r_s_ = 0.60; *p* < 0.0001) (Figure 1B), the DPA (r_s_ = 0.53; *p* = 0.0005) (Figure 1C) and DHA (r_s_ = 0.66; *p* < 0.0001) (Figure 1D).

**Figure 2:**
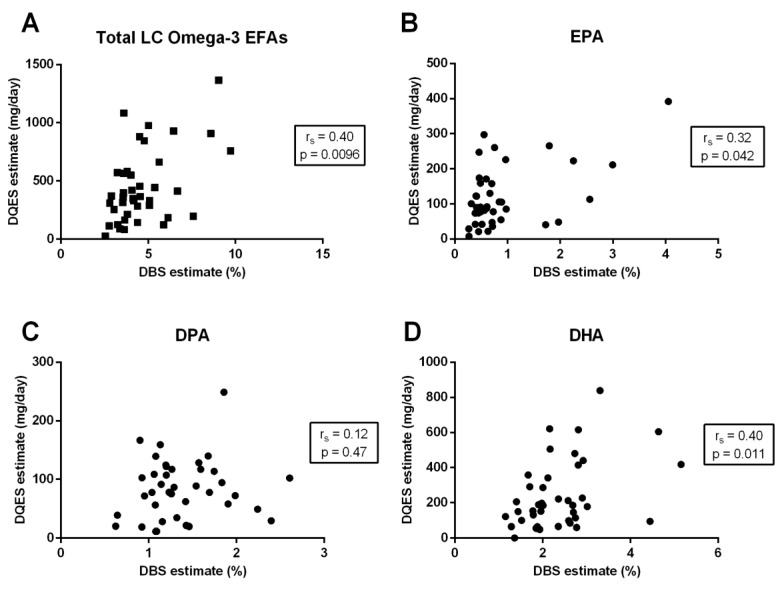
The relationship between total long-chain (LC) omega-3 essential fatty acids (EFAs) percentage (%) present in erythrocytes, measured using dried blood spot (DBS) biomarkers, and the dietary LC omega-3 EFA intake estimated using the Dietary Questionnaire for Epidemiology Studies (DQES) v3.2 for (**A**) total LC omega-3 EFAs, (**B**) eicosapentaenoic acid (EPA), (**C**) docosapentaenoic acid (DPA), and (**D**) docosahexaenoic acid (DHA). Correlation coefficients are calculated using the Spearman’s correlation coefficients (r_s_).

Comparing the DQES (v3.2) to the DBS assay, there was a moderate correlation for both total long-chain omega-3 EFAs (r_s_ = 0.40; *p* = 0.0096) (Figure 2A) and DHA (r_s_ = 0.40; *p* = 0.011, Figure 2D) and a weak, positive correlation for EPA (r_s_ = 0.32; *p* = 0.042) (Figure 2B). There was no significant relationship between the DQES (v3.2) and DPA levels measured using the DBS test (r_s_ = 0.12; *p* = 0.47) (Figure 2C).

**Figure 3:**
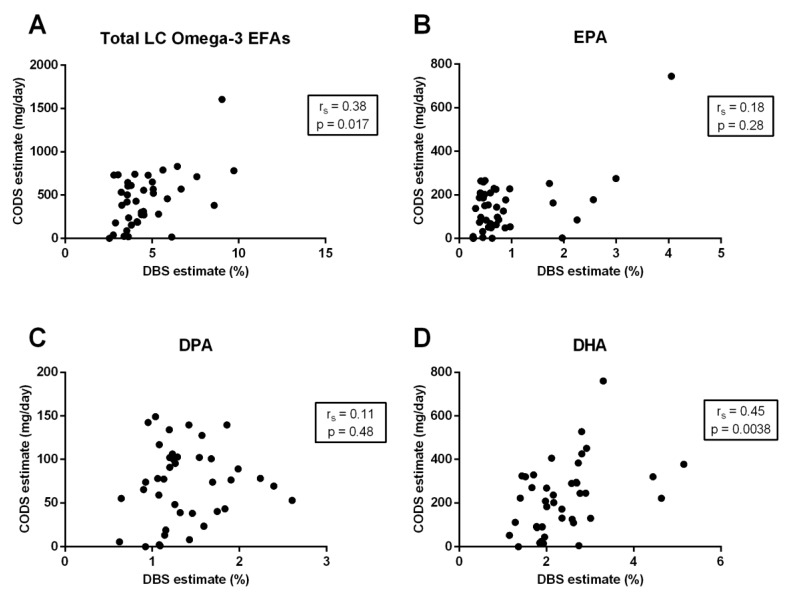
The relationship between total long-chain (LC) omega-3 essential fatty acids (EFAs) percentage (%) present in erythrocytes, measured using dried blood spot (DBS) biomarkers, and the dietary LC omega-3 EFA intake estimated using the Clinical Omega-3 Dietary Survey (CODS) for (**A**) total LC omega-3 EFAs, (**B**) eicosapentaenoic acid (EPA), (**C**) docosapentaenoic acid (DPA), and (**D**) docosahexaenoic acid (DHA). Correlation coefficients are calculated using the Spearman’s correlation coefficients (r_s_).

Comparing the CODS to the DBS fatty acid analysis, there was a moderate positive correlation between the two methods for total long-chain omega-3 EFAs (r_s_ = 0.38; *p* = 0.017) (Figure 3A) and DHA (r_s_ = 0.45; *p* = 0.0038) (Figure 3D), and a weak correlation for EPA (r_s_ = 0.18; *p* = 0.28) (Figure 3B), but no significant correlation for DPA (r_s_ = 0.11; *p* = 0.48) (Figure 3C).

**Figure 4:**
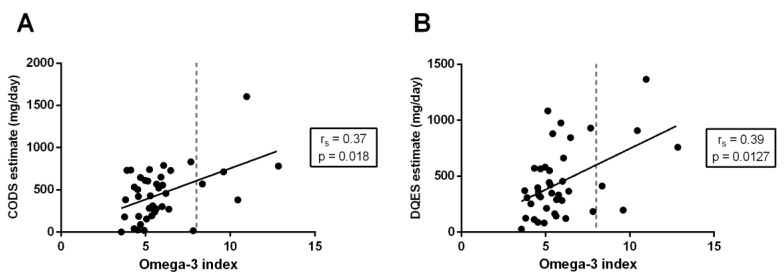
The relationship between the overall Omega-3 index, calculated as the total percentage (%) of EPA and DHA present in erythrocytes, and estimated using the dried blood spot (DBS) biomarker, and the dietary estimate of total long-chain omega-3 intake using (**A**) the Clinical Omega-3 Dietary Survey (CODS) and (**B**) the Dietary Questionnaire for Epidemiology Studies (DQES) v3.2. Correlation coefficients are calculated using the Spearman’s correlation coefficients (r_s_).

For total long-chain omega-3 EFA intake, the relationship between both dietary methods (i.e., CODS and DQES) and the DBS Omega-3 index, which is a measure of the total EPA and DHA present in erythrocyte phospholipid membranes, was considered. There was a moderate, positive correlation between both the CODS (r_s_ = 0.37; *p* = 0.018) (Figure 4A) and DQES (r_s_ = 0.39; *p* = 0.013) (Figure 4B) questionnaires compared to the Omega-3 index estimated using the DBS assay. 

### 3.4. Method of Triads Analysis

Table 4 summarizes the validity coefficient (ρ) for each of the methods used to estimate the dietary omega-3 EFAs (CODS, DQES, and DBS), relative to an estimated true intake (T), calculated using the method of triads [43]. 

The CODS obtained high validity coefficients for estimating each of total long-chain omega-3 EFAs (ρ = 0.77, 95% CI: 0.31 to 0.98); DHA (ρ = 0.86, 95% CI: 0.54 to 0.99); and DPA (ρ = 0.72, 95% CI: 0.14 to 0.97). A moderate validity coefficient was obtained for estimating EPA (ρ = 0.57, 95% CI: 0.21 to 0.93).

The DQES obtained high validity coefficients for estimating total long-chain omega-3 EFAs (ρ = 0.83, 95% CI: 0.39 to 0.98); DHA (ρ = 0.77, 95% CI: 0.39 to 0.97); and DPA (ρ = 0.73, 95% CI: 0.16 to 0.97). A Heywood case was met for the validity coefficient estimate for EPA (ρ = 1.04), and thus the validity coefficient was set to 1.0 (95% CI: 0.39 to 1.00).

For the DBS assay, moderate validity coefficients (relative to the estimated true intake T) were obtained for estimating each of total long-chain omega-3 EFAs (ρ = 0.49, 95% CI: 0.12 to 0.73); DHA (ρ = 0.52, 95% CI: 0.21 to 0.74); and EPA (ρ = 0.31, 95% CI: 0.07 to 0.65). A low validity coefficient was obtained for estimating DPA (ρ = 0.16, 95% CI: 0.03 to 0.50).

### 3.5. Bland–Altman Analysis

**Figure 5:**
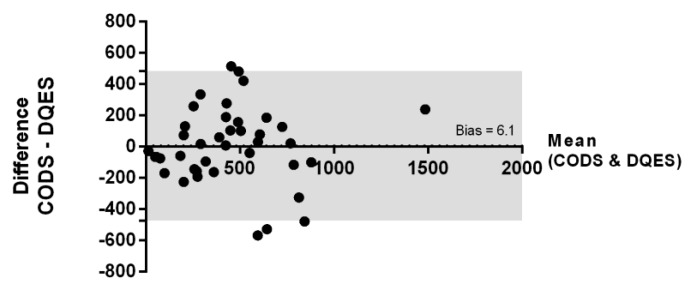
Bland–Altman plot comparing the estimated total daily intake of long-chain omega-3 EFAs (mg/day), measured using the CODS and DQES. The dotted line shows the bias (6.1 mg/day) for the comparison between the two methods, which was not statistically significant (95% CI: −32.5 to 44.7 mg/day). The grey shaded area highlights the limits of agreement (LoA).

The inter-method agreement was assessed using a Bland–Altman analysis (Figure 5) for the CODS- and the DQES-derived estimates for total daily long-chain omega-3 EFA intake (mg/day). Regression analysis indicated the absence of a significant inter-variable relationship across the spectrum of quantified values (*p* = 0.97). There was no significant global bias (mean difference) between these two assessment methods (mean ± standard error: 6.1 ± 38.6 mg/day, *p* > 0.05).

## 4. Discussion

This preliminary validation study shows that a novel FFQ, the CODS, is a simple, valid tool for assessing long-chain omega-3 EFA intake in Australian adults. The CODS, which considers foods containing the highest concentrations of long-chain omega-3 EFAs, extracted from an Australian food compositional database (AUSNUT 2011–2013) [34], only requires a few minutes to complete. The dietary estimates derived from the CODS correlated moderately well with long-chain omega-3 fatty acid intake quantified using the comprehensive, validated DQES (v3.2), as well as the systemic fatty acid profiles derived from dried blood spot analyses. Given that the CODS is straightforward to use, and can be rapidly completed in clinical settings, we propose that this survey can be applied clinically to estimate patients’ long-chain omega-3 EFA intake, particularly in eye care settings where no similar tools currently exist. Therefore, this may provide information to inform clinical advice with respect to the sufficiency of a patient’s estimated intake of dietary long-chain omega-3 EFAs, and thus any potential recommendations surrounding dietary modification and/or supplementation.

Knowledge of a patient’s baseline omega-3 status is essential to develop informed, best-practice clinical recommendations relating to the relative appropriateness of dietary adjustment(s) and/or supplementation. Biomarker-based assessments of systemic omega-3 EFA levels, typically derived from subcutaneous adipose or blood assays, although accurate, are costly and time consuming, and therefore are not routinely applied in clinical settings. In addition, these types of biomarker assessments may not be readily available to all clinicians who require knowledge of patients’ omega-3 fatty acid intake to provide informed clinical care. For example, as major providers of primary eye care, optometrists frequently provide clinical care to individuals who are at risk of, or who have existing, eye conditions where the natural history of the disease may be influenced by omega-3 EFA intake. Recent research demonstrates that eye care clinicians have identified a need for validated clinical tools to assess the degree of dietary omega-3 fatty acid sufficiency for their patients [46]. Furthermore, when attending eye examinations, there is an expectation among patients that the provision of comprehensive clinical care includes an assessment and relevant evidence-based advice surrounding dietary risk factors for ocular disease [47]. A major limitation with generic clinical recommendations regarding omega-3 EFA intake (e.g., to eat oily fish at least twice a week) is that such advice does not take into consideration the substantial variability of omega-3 fatty acid concentrations across different species of fish. For example, a 100 g serving of salmon provides approximately seven-fold more long-chain omega-3 EFAs than that of 100 g of lean fish, such as whiting [48]. In this regard, tailored questionnaires that are designed to specifically assess EFA intake may have the capacity to more accurately capture dietary intakes for specific nutrients (e.g., fatty acids) compared with generic questionnaires (i.e., questionnaires that assess total dietary intake but do not consider the specific fatty acid sources) [49].

Several FFQs have been developed to assess dietary omega-3 intake [21,50], which range from long [27] to short [29,30,31] in length. Questionnaires may perform differently in different geographic locations and patient populations, and as such, instruments should be validated by recruiting participants who are representative of the primary target population [51]. In terms of relatively short, clinically applicable FFQs, Sublette et al. developed and validated a 21-item questionnaire, which took approximately five minutes to complete, administered with a sample of 61 US adults with and without major depressive disorders. [31]. Dahl et al. developed and validated another brief, 10-minute questionnaire, administered with a sample of healthy Norwegian population [29]. A nine-item FFQ was developed based on the 2005 Canadian Nutrient File of Health Canada, conducted in a sample of women with low marine food intakes and with psychological stress [30]. It showed poor agreement with an Australian-based FFQ, when administered in an Australian population, however, estimates of the nutritional intake improved when nutritional composition data were replaced with Australian equivalents [32]. 

We validated the CODS relative to both an erythrocyte biomarker and a previously validated food frequency questionnaire (DQES v3.2). Nutritional information provided by the DQES differed from the CODS as it comprehensively considers all food groups available in Australia, in order to create a full dietary profile of consumed nutrients. In contrast, the CODS only assesses foods that are rich in omega-3 EFAs. Information relating to long-chain omega-3 EFA intake was extracted from the DQES based upon food compositional data from the NUTTAB (2010) database [39], whereas dietary estimates for the CODS were extracted from the independent AUSNUT (2011–2013) database [34]. Other potential dietary reference methods included 24-h food recalls, and short-term and long-term food diaries. These techniques provided an alternative, but not necessarily more accurate, method of assessing dietary intake [51]. The limitation of short-term food diaries is the day-to-day variation of foods consumed, and as such, a single observation may provide a poor measure of overall dietary intake [21]. Furthermore, although the food records provided a precise measure of dietary habits and portion sizes over the short monitoring period (e.g., seven days), bias may be induced as dietary behaviors of a participant may be influenced over the capture period [52]. 

The limitation of not capturing a baseline omega-3 status, which in practice may inform clinical recommendations, is similar to the confound of not quantifying this parameter in omega-3 EFA intervention trials, in order to consider this factor in the evaluation of therapeutic efficacy. For example, numerous clinical studies have sought to investigate the therapeutic efficacy of omega-3 EFA supplements for treating dry eye disease, with many reporting apparently differing results [53,54,55,56]. However, as recently reported in a Cochrane systematic review [57] of 34 randomized controlled trials assessing the effect(s) of oral omega-3 and/or omega-6 supplements on dry eye symptoms and signs, the vast majority of studies to date have not surveyed (through food questionnaires) or quantified (e.g., through blood testing) baseline systemic fatty acid levels. It is critical to have an understanding of basal levels, as individuals already achieving sufficient levels of PUFAs from food sources may not demonstrate the same response to a given dose of fatty acid supplementation as those with a diet deficient in some, or all, PUFAs [58,59]. The assessment of post-treatment omega-3 status is also a means of evaluating participant compliance (in addition to other measures such as returned capsule counts, compliance diaries, etc.), however, in this systematic review, it was only assessed in two of the included trials [54,55]. While the quantification of baseline omega-3 EFA levels is recommended as a standard for cardiovascular trials [58], currently, there is no such guidance in relation to ocular studies. If the cost of biological assays is prohibitive, the findings of our study suggest that the CODS may be a relevant surrogate marker for estimating dietary intake of long-chain omega-3 EFAs. 

A potential advantage of using methods based on surveys, rather than biological biomarkers, to assess omega-3 EFA intake is the ability to disambiguate fatty acids consumed from food sources and nutritional supplements. This is of particular relevance to the clinical recommendations for omega-3 EFAs in the context of eye disease, where the source of omega-3 EFAs may influence clinical outcomes. For example, several epidemiological studies have shown that a high (food-sourced) dietary intake of omega-3 fatty acids is associated with a significantly reduced risk of developing age-related macular degeneration (AMD) [8,9], and a decreased risk of AMD progression in individuals with the established disease [10,11,12]. However, perhaps counter intuitively, a Cochrane collaboration systematic review of randomized controlled trial evidence reported that the use of omega-3 fatty acid nutritional supplements, for a follow-up period of up to five years, did not reduce the incidence of progression to late-stage AMD or the development of moderate-to-severe vision loss, compared to placebo supplementation [60]. There are currently no randomized controlled trials on dietary omega-3 EFA supplementation for the primary prevention of AMD. Therefore, whole food sources containing high levels of long-chain omega-3 EFAs have been shown to be retinoprotective, whereas, omega-3 supplementation does not appear to confer the same benefit. The specific mechanism(s) underlying the benefits of omega-3 EFAs in AMD have not been established, but likely derive from the anti-inflammatory and/or anti-oxidative effects of omega-3 EFAs [60], and the potential interaction of these fatty acids with other nutrients found in whole foods rich in these components.

The Omega-3 index is a measure of the relative concentration of EPA and DHA in erythrocyte membranes, and it is considered an acceptable marker for evaluating the risk of coronary heart disease [24]. An association has been reported between Omega-3 indices <4% and a high incidence of cardiovascular disease; shifting the Omega-3 index from 4% to 8% is also estimated to reduce the relative incidence of fatal coronary heart disease by 30% [61]. In the present study, we compared the DQES and CODS (survey-based) estimates of daily long-chain omega-3 EFA intake to the Omega-3 index quantified using the DBS biomarker assay. Total long-chain omega-3 EFA intake, estimated with both the CODS and DQES v3.2, showed a moderately strong, positive correlation with the Omega-3 index (CODS: r_s_ = 0.39, *p* = 0.013; DQES v3.2: r_s_ = 0.37, *p* = 0.018). Of note, on the basis of interpolation of the data shown in Figure 4, targeting a desired Omega-3 index of 8% [61] corresponds to an intake of ~600 mg/day of long-chain omega-3 EFAs, as estimated using the CODS. Consistently, this estimate agrees with NHMRC SDT recommendations for lowering chronic disease risk [15,19].

In general, the accuracy of dietary surveys depends on the recall ability of the participant and the accuracy and availability of nutritional composition data in the geographical region [33]. We acknowledge a limitation of the CODS is that it does not consider the conversion of ALA and short-chain omega-3 fatty acid to long-chain metabolites, or the consumption of omega-6 fatty acids, being the other major class of PUFAs. Foods containing ALA from nutrition composition databases were not considered due to the heterogeneity in ALA concentrations reported across foods in the same groups (e.g., the amount of ALA present in margarine varies from 1 g/100 to 8 g/100 g, depending on the brand and type i.e., monounsaturated or polyunsaturated) [34], and the additional time that would have been required to complete a questionnaire that incorporated these food sources. The decision to omit ALA estimates from the CODS was also based on the known efficiency of conversion of short- to long-chain omega-3 fatty acid forms *in vivo*, which has been reported to range from 4–20% for EPA and estimated at 5–9% for both DPA and DHA [62,63,64]. This conversion is further reduced in the presence of increasing circulating levels of omega-6 EFAs [62]. Long-chain omega-6 EFAs also competitively inhibit the incorporation of omega-3 EFAs into phospholipid membranes [65]. The poor validity coefficient between DPA intake estimated from survey methods and a biological biomarker has also been observed in other studies in the same region [27,41], and may be related to the selective uptake of DPA in tissues [66,67]. DPA, being an intermediate metabolite, is also highly interconvertible with EPA and less readily metabolized to DHA [66,67]. These factors may contribute to the lack of correlation between the DPA estimate using CODS and the DBS biomarker assay. Nevertheless, we observed a significant correlation between the CODS and the DBS biomarker assay, for total long-chain omega-3 fatty acids, DHA, and EPA. Further validation of the CODS, in particular within a larger population of patients in clinical practice, would be of value to confirm the generalizability of these findings.

In this preliminary investigation, we demonstrated that the CODS is a potentially useful tool for assessing long-chain omega-3 intake, validated in a population of Australian adults, factoring in both food sources and dietary supplementation. In addition, the CODS estimated daily intake of long-chain omega-3 EFAs was moderately well correlated to the Omega-3 index, which is a validated marker for cardiovascular disease risk. We propose that the CODS could provide a rapid, non-invasive tool (as a no-cost alternative application as compared with more costly investigations) for evaluating the relative sufficiency of a patient’s dietary omega-3 EFA intake in a clinical setting. Future directions will include repeatability assessment and validation of the CODS against another well-controlled dietary reference method (e.g., a multiple-day food record), in a larger, homogenous population of participants. These additional investigations will be of value for strengthening the rigor of the CODS, and deriving further data to support its utility for implementation in eye care practice.

## Figures and Tables

**Table 1:** Comparison of the median and inter-quartile range (IQR) values for long-chain omega-3 fatty acid intake, for the CODS and DQES v3.2 questionnaires, in the study cohort.

	CODS (mg/day)			DQES v3.2 (mg/day)			*p*-value
	Median	IQR		Median	IQR		
		p25	p75		p25	p75	
Total LC omega-3 EFAs	443.7	204.1	650.0	362.6	200.7	577.9	0.83
EPA (20:5-n3)	140.5	55.9	209.2	91.4	49.9	168.0	0.37
DPA (22:5-n3)	75.2	39.3	102.2	77.8	41.3	116.2	0.41
DHA (22:6-n3)	221.7	95.8	320.2	184.9	98.2	353.7	0.98

Abbreviations: CODS, Clinical Omega-3 Dietary Survey; DHA, docosahexaenoic acid; DPA, docosapentaenoic acid; DQES, Dietary Survey for Epidemiology Studies v3.2; EFA, essential fatty acid; EPA, eicosapentaenoic acid; IQR, inter-quartile range; LC, long-chain; p25, 25th percentile; p75, 75th percentile.

**Table 2:** Concentration of long-chain omega-3 EFAs present in erythrocytes for the study cohort.

	Median	IQR	
		p25	p75
Total LC omega-3 EFAs (%)	4.15	3.58	5.30
EPA (20:5-n3) (%)	0.60	0.45	0.88
DPA (22:5-n3) (%)	1.25	1.08	1.66
DHA (22:6-n3) (%)	2.16	1.80	2.77
Omega-3 index	5.32	4.58	6.17

Abbreviations: EFA, essential fatty acid; EPA, eicosapentaenoic acid; DHA, docosahexaenoic acid; DPA, docosapentaenoic acid; IQR, inter-quartile range; LC, long-chain; total LC omega-3 EFAs, DHA + DPA + EPA; p25, 25th percentile; p75, 75th percentile.

**Table 3:** Relationships between the long-chain omega-3 EFA quantification methods, calculated using the Spearman’s correlation coefficient (r_s_).

	CODS vs. DQES		DBS vs. DQES		DBS vs. CODS	
	r_s_	*p*-value	r_s_	*p*-value	r_s_	*p*-value
Total LC omega-3 EFAs	0.64	<0.0001	0.40	0.0096	0.38	0.017
EPA (20:5-n3)	0.60	<0.0001	0.32	0.042	0.18	0.28
DPA (22:5-n3)	0.53	0.0005	0.12	0.47	0.11	0.48
DHA (22:6-n3)	0.66	<0.0001	0.40	0.011	0.45	0.0038

Abbreviations: CODS, Clinical Omega-3 Dietary Survey; DBS, dried blood spot; DHA, docosahexaenoic acid; DPA, docosapentaenoic acid; DQES, Dietary Survey for Epidemiology Studies v3.2; EFA, essential fatty acid; EPA, eicosapentaenoic acid; LC, long-chain; total LC omega-3 EFAs, DHA + DPA + EPA.

**Table 4:** Validity coefficients (ρ) calculated using the method of triads for each of the methods vs. the estimated true intake (T) for each of the long-chain omega-3 EFAs.

	CODS Validity Coefficient vs. T [ρQT] (95% CI)	DQES Validity Coefficient vs. T [ρRT] (95% CI)	DBS Validity Coefficient vs. T [ρBT] (95% CI)
Total LC omega-3 EFAs	0.77 (0.31–0.98)	0.83 (0.39–0.98)	0.49 (0.12–0.73)
EPA (20:5n-3)	0.57 (0.21–0.93)	1.00 * (0.39–1.00)	0.31 (0.07–0.65)
DPA (22:5n-3)	0.72 (0.14–0.97)	0.73 (0.16–0.97)	0.16 (0.03–0.50)
DHA (22:6n-3)	0.86 (0.54–0.99)	0.77 (0.39–0.97)	0.52 (0.21–0.74)

* Validity coefficients >1 were set to 1.00 (Heywood cases). Abbreviations: CI, confidence interval; CODS, Clinical Omega-3 Dietary Survey; DBS, dried blood spot; DHA, docosahexaenoic acid; DPA, docosapentaenoic acid; DQES, Dietary Survey for Epidemiology Studies v3.2; EFA, essential fatty acid; EPA, eicosapentaenoic acid; LC, long-chain; total LC omega-3 EFAs, DHA + DPA + EPA; Q, questionnaire; R, reference method; T, true intake.

## Supplementary Materials

The following are available online at https://www.mdpi.com/2072-6643/11/4/817/s1, Figure S1: The Clinical Omega-3 Dietary Survey, Figure S2: Diagram adapted from Ocke & Kaaks, to describe the method of triads [43].
