# Supplementary material for: Preliminary Validation of a Food Frequency Questionnaire to Assess Long-Chain Omega-3 Fatty Acid Intake in Eye Care Practice
Source: Nutrients. 2019 Apr 11;11(4):817. doi: 10.3390/nu11040817 (PMC6521311; doi:10.3390/nu11040817)
Supplement: Supplementary file 1 [file nutrients-11-00817-s001.pdf]

Clinical Omega-3 Dietary Survey

How many times have you eaten fish or shellfish in the past week?

❒ 0 times ❒ 1-3 times ❒ More than 3 times

Over the past 3 months, about how often have you eaten fish or shellfish in any form?

❒ Never ❒ __________ times per *(please circle)* Day/Week/Month

Each time you ate fish or shellfish, how much did you eat?
❒ Less than 50 g or half a fillet or less than 4 pieces/1-2 hand roll of sushi
❒ 100 g or about 1 small fillet or 8 pieces of sushi/3 hand rolls
❒ 150 g or 1 large fillet or 12 pieces of sushi/4 hand rolls
❒ 200 g+ or more than 2 fillets or 16 pieces of sushi/6 hand rolls

Please mark the food(s) you eat regularly (i.e., at least once per month) over the last three (3) months and indicate:

- your average portion for <u>EACH</u> serve
- on average, how often you eat that type of food

90 g = approximately the size of your palm or a deck of cards.

Seafood

❒	Anchovy	.... g	.... times a week/month
❒	Eel	.... g	.... times a week/month
❒	Mussel	.... g	.... times a week/month
❒	Oyster	.... g	.... times a week/month
❒	Scallop	.... g	.... times a week/month
❒	Prawn	.... g	.... times a week/month
❒	Squid	.... g	.... times a week/month

Fish

❒	Barramundi	.... g	.... times a week/month
❒	Bassa (Basa)	.... g	.... times a week/month
❒	Hoki	.... g	.... times a week/month
❒	Blue Eye Trevalla	.... g	.... times a week/month
❒	Kingfish/Trevally	.... g	.... times a week/month
❒	Herring	.... g	.... times a week/month
❒	Mackerel	.... g	.... times a week/month
❒	Salmon	.... g	.... times a week/month
❒	Sardine	.... g	.... times a week/month
❒	Silver perch	.... g	.... times a week/month
❒	Snapper	.... g	.... times a week/month
❒	Swordfish	.... g	.... times a week/month
❒	Trout	.... g	.... times a week/month
❒	Tuna	.... g	.... times a week/month
❒	Whiting	.... g	.... times a week/month
❒	Fish fingers	.... g	.... times a week/month
❒	Fish (white flesh)	.... g	.... times a week/month

Other meats

❒ Bacon ……………. g …………… times a week/month

❒ Beef ……………. g …………… times a week/month

❒ Kangaroo ……………. g …………… times a week/month

❒ Lamb ……………. g …………… times a week/month

❒ Salami ……………. g …………… times a week/month

❒ Pork ……………. g …………… times a week/month

❒ Egg (per egg) ……….. eggs …………… times a week/month

Omega-3 Supplements

❒ Omega-3/Fish oil (please specify Brand/type/dose) ………. g/caps/TBS

…………………………………………………………………………… ……….. times a day/week

Figure S1: Clinical Omega-3 Dietary Survey (CODS)

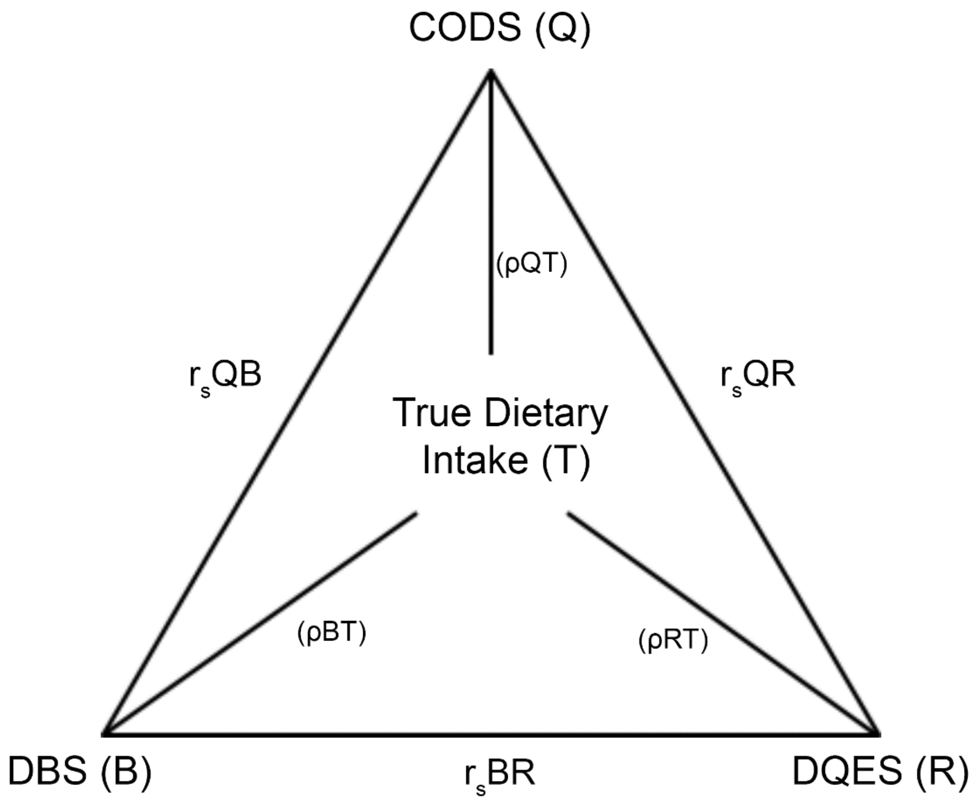

Figure S2: Diagram adapted from Ocke & Kaaks, to describe the 'method of triads' [43] used to estimate the validity coefficient (ϱ) for long-chain omega-3 dietary intake estimated using the questionnaire (Q), Clinical Omega-3 Dietary Survey (CODS); a reference marker (R), the Dietary Questionnaire for Epidemiology Studies (DQES) v3.2; and a biomarker (B), the dried blood spot (DBS) test. Spearman's correlation coefficient (r_s) was used to estimate the correlation coefficient between each method. Validity coefficients (ϱ) were calculated using methods as described by Ocke & Kraaks for each of the reference methods against an estimated true intake (T) [43].
